# Technologieauswahl im DigitalPakt: Wie werden Entscheidungen im Bildungssektor getroffen?

**DOI:** 10.1365/s40702-021-00751-x

**Published:** 2021-06-28

**Authors:** Lukas Petry, Sebastian Lins, Scott Thiebes, Ali Sunyaev

**Affiliations:** Fakultät für Wirtschaftswissenschaften, Institut für Angewandte Informatik und Formale Beschreibungsverfahren (AIFB), Karlsruher Institut für Technologie, Karlsruhe, Deutschland

**Keywords:** DigitalPakt Schule, Technologieauswahl, Bildungssektor, Öffentlicher Bereich, Entscheidungskriterien, DigitalPakt Schule, Technology selection, Educational system, Public sector, Decision-making factors

## Abstract

Der DigitalPakt Schule soll das deutsche Bildungswesen flächendeckend modernisieren, zeigt jedoch bisher Anlaufschwierigkeiten. Die bereitgestellten Fördermittel werden zögerlich abgerufen und die Kritiker:innen des Programms werden immer lauter. Diese Arbeit setzt genau an diesem Punkt an und diskutiert Ursachen und Einflüsse, die dazu beitragen, dass verfügbare Technologien bisher von Schulen nicht oder nur unzureichend angenommen werden. Diese Diskussion ist insbesondere wichtig, da das Schulsystem des Landes zurzeit durch die pandemie-bedingte Krise im Fokus steht. Dafür wurden semi-strukturierte Expert:inneninterviews mit Schulvertreter:innen durchgeführt und mit Hilfe von wissenschaftlichen Kodierungsmethoden analysiert. Die Ergebnisse geben einen Einblick in den Technologieauswahlprozess, um herauszufinden, wie Auswahlentscheidungen im Bildungssektor getroffen werden, und zu verstehen, warum der öffentliche Bereich eine Sonderstellung einnimmt und nicht so funktioniert, wie ein wirtschaftsdenkendes Unternehmen.

## Abstract (en)

The German “*DigitalPakt Schule*” is a financial support package by the state, intended to modernize the educational system. Despite its promising vision, the realization slowed down and critical voices arose. The stagnation in this process is a reason why the educational system in Germany currently struggles during the COVID-19 crisis. To understand the reasons and problems during the selection of technologies suitable for schools, a closer look into the technology selection process is needed, particularly because the public sector is not driven by economic thoughts and therefore its processes differ from traditional corporate models. For this research, semi-structured expert interviews are conducted and analyzed with scientific coding methods. The goal of this study is to make the process more transparent and identify the factors that impact technology selection in schools. The identification of those factors portrays the unusual nature of the educational system and supports the development of an efficient approach to its digitalization.

## Einleitung

*Von Theodor Fontane stammt der Satz „Alles Alte, soweit es Anspruch darauf hat, sollten wir lieben, aber für das Neue sollen wir […] leben.“ In der Frage der Digitalisierung halte ich es wie Fontane. Obwohl vor 200 Jahren, zu Lebzeiten Fontanes, die Digitalisierung natürlich noch kein Thema war. Der darauffolgende Satz lautet: „Und vor allem sollen wir, […], den großen Zusammenhang der Dinge nie vergessen.“ In großen Zusammenhängen zu denken, ist gerade bei der Digitalisierung unerlässlich*.

Mit diesen Worten eröffnete Bildungsministerin Anja Karliczek, den sechsten Tag der ökonomischen Bildung NRW am 7. März 2019. Zwei Monate darauf trat der bis dahin oft diskutierte DigitalPakt Schule in Kraft. Diskutiert wurde vor allem die damit verbundene Änderung des deutschen Grundgesetzes, das eine Finanzierung des Ausbaus von Schulen durch den Bund bis dato nicht vorgesehen hatte, da Bildungspolitik durch die einzelnen Bundesländer geregelt wird.

Deutsche Bildungseinrichtungen sind, bezogen auf den Stand der Modernisierung, im internationalen Vergleich auf einem niedrigen Niveau (Eickelmann und Labusch 2019), was sich in der Infrastruktur der Schulen widerspiegelt und besonders durch die krisenbedingten Schulschließungen im Jahr 2020 bis heute mediale Aufmerksamkeit erhielt. In den durch die Corona-Pandemie hervorgetretenen Zeiten von Wechsel- und Fernunterricht wird die Wichtigkeit einer digitalen Schule auf beunruhigende Weise deutlich. Die Bundesregierung will den Rückstand in der Digitalisierung von Schulen aufholen und stellt durch den sogenannten „DigitalPakt Schule“ zusätzliche Finanzmittel für die Digitalisierung der Bildungsbranche zur Verfügung.

Allerdings wurde ein Großteil der bereitgestellten Fördermittel bisher nicht abgerufen (Brandau und Pauly 2020), weshalb die Frage aufkommt, was die Gründe dafür sind, dass die Beantragung der Fördergelder und der neuen Technologien länger dauert als erhofft. Erste Kommentare dazu verweisen auf lähmende Prozesse, fehlende Umsetzung durch die Ländergremien und Unsicherheiten in den Schulen (bspw. Wahl 2019). Dieses unübersichtliche Gefüge gilt es zu entwirren und die Frage zu beantworten, welche Technologien zum Einsatz kommen sollen. Aufgrund wenig anwendbarer Forschung und neuen Rahmenbedingungen durch den DigitalPakt lässt sich der Entscheidungsprozess für die Anschaffung neuer Infrastruktur im Bildungsbereich noch nicht präzise darstellen. Ziel dieser Arbeit war es zu untersuchen, wie man den Prozess der Technologieauswahl transparenter darstellen kann. Es wird explorativ erforscht, welche Kriterien den Auswahlprozess von Technologien im öffentlichen Bereich im Kontext des DigitalPakts beeinflussen. Dabei wird auch deutlich, wie er sich von bisherigen Wirtschaftsprozessen abhebt. Das Hauptziel der Arbeit besteht demnach in der Beantwortung der Frage: *Wie gestaltet sich der Technologieauswahlprozess im Bildungsbereich im Kontext des DigitalPakt Schule?*

## Der DigitalPakt Schule

Der DigitalPakt Schule ist ein Unterstützungspaket der Bundesrepublik Deutschland zur Förderung der digitalen Infrastruktur von Bildungseinrichtungen (BMBF 2019). Die Vereinbarung zwischen dem Bundesministerium für Bildung und Forschung (BMBF) und den Bildungsministerien der Länder umfasst ursprünglich eine Förderung von fünf Milliarden Euro über einen Zeitraum von fünf Jahren – 2019 bis 2024, bereitgestellt durch das BMBF. Die exakten Richtlinien, nach denen die Mittel beantragt werden und wie der Prozess im Detail abläuft, legen die einzelnen Bundesländer individuell für sich fest. Die Förderanträge basieren auf sogenannten Medienkonzepten, pädagogisch-didaktischen Konzepten zur digitalen Schultransformation, die jede Schule individuell erstellen und dann beim Schulträger einreichen muss, um Fördergelder beantragen zu können. Hat der Schulträger, meistens die Kommune, das Konzept geprüft, stellt dieser den Antrag beim Land. Dort wird der Förderantrag auf die Einhaltung der Förderrichtlinien geprüft. Nach Genehmigung können vom Schulträger öffentliche Ausschreibungen erstellt werden, um die Umsetzung des Konzepts zu initiieren und durchzuführen.

Die durch den DigitalPakt garantiert förderfähigen Technologien betreffen den Ausbau des schulischen WLAN sowie die damit verbundene Verkabelung und Vernetzung der Schulgebäude. Außerdem werden stationäre, digitale Endgeräte gefördert sowie eingeschränkt mobile Endgeräte, sofern eine funktionierende Infrastruktur verfügbar ist und die Kosten einen gewissen Grenzbetrag nicht überschreiten. Im Zuge der COVID-19 Pandemie und den damit verbundenen Schulschließungen wurde die Notwendigkeit solcher Fördermaßnahmen zur Aufrechterhaltung der Souveränität des deutschen Bildungssystems verdeutlicht. Deshalb hat sich die Bundesregierung im September 2020 darauf geeinigt, weitere eineinhalb Milliarden Euro für kurzfristige Sofortmaßnahmen zur Stärkung des Schulsystems zur Verfügung zu stellen (BMBF 2020).

## Forschungsmethodisches Vorgehen

Die vorliegende Forschungsarbeit wurde in Zusammenarbeit mit der LANCOM Systems GmbH durchgeführt und basiert auf einem qualitativen Vorgehen, da der theoretische Erkenntnisstand bisher nicht ausgereift ist und hiermit explorativ neue Erkenntnisse identifiziert werden sollen. Insbesondere sollen Kriterien durch Expert:inneninterviews identifiziert werden, welche den Technologieauswahlprozess im Bildungssektor im Kontext des DigitalPakts Schule beeinflussen. Die primäre Methode der Datenerhebung sind semi-strukturierte Interviews nach Myers (2009).

### Akquise der Interviewpartner:innen

Die Zielgruppe der Interviews bildet der Personenkreis von verantwortlichen Entscheidungsträger:innen bei Schulträgern sowie bei beratenden Organisationen aus unterschiedlichen Bundesländern. Interviewpartner:innen wurden durch persönliche Kontakte sowie Kaltakquise kontaktiert und zur Teilnahme an der Studie eingeladen. Insgesamt erklärten sich fünf Experten:innen bereit, an der Studie teilzunehmen. Viele weitere Experten:innen wurden angefragt, mussten jedoch aufgrund der aktuell sehr hohen Auslastung ihre Teilnahme absagen. Durch die langjährige Mitarbeit der Befragten in der relevanten Branche und eine starke Einbindung in die Prozesse konnten fundierte Einblicke erwartet werden. In Tab. 1 sind die fünf befragten Personen anonymisiert dargestellt.

**Tab. 1:** Zusammenfassung der Interviewpartner:innen

ID	Organisation (Bundesland)	Position	Jahre in der Organisation	Altersgruppe	Einbindung in Entscheidungsprozesse	Dauer des Interviews
I1	Schulverwaltungsamt (NW)	Leitung	40	60–70	Hoch	71 min
I2	Schulverwaltungsamt (BY)	Leitung/Vorsitz	7/3	40–50	Hoch	54 min
I3	Schul- & Medienberatung (NI)	Leitung	8	40–50	Hoch	53 min
I4	Seminar für Ausbildung und Fortbildung der Lehrkräfte (BW)	Ausbildung & Netzwerkadministration	15	40–50	Hoch	91 min
I5	Schul- & Medienberatung (HE)	Leitung	9	30–40	Hoch	55 min

### Interviewdurchführung

Zur Vorbereitung der Interviews wurde ein Interviewleitfaden erstellt und mit weiteren Forscher:innen validiert. Fokus der Interviews waren der Umgang und die Erfahrungen der Befragten mit dem DigitalPakt Schule, und den damit verbundenen Technologieauswahl- und Entscheidungsprozessen im öffentlichen Bereich. Der Interviewleitfaden wurde zwischen jedem Interview überprüft und mithilfe der bisher gewonnen Erkenntnisse erweitert und abgeändert, um neue Aspekte in die darauffolgenden Gespräche einzubringen und in Beachtung des theoretischen Samplings die potenziellen Kriterien zu diskutieren (Urquhart et al. 2010). Die Interviews dauerten im Durchschnitt jeweils 65 min. Sie wurden aufgrund der zur Zeit der Forschung bestehenden Kontaktbeschränkungen digital durchgeführt und aufgezeichnet. Die Aufzeichnungen wurden anschließend anonymisiert und transkribiert, um eine Auswertung zu ermöglichen.

### Analyse der Interviewdaten

Zur Beantwortung der Forschungsfrage und Analyse der Interviewdaten wurde ein mehrschichtiges Kodierungsverfahren, angelehnt an Corbin und Strauss (2015), durchgeführt. Dabei wurden die erhobenen Interviewdaten mit Hilfe der Software Atlas.ti 7 in mehreren Schritten kodiert:

*Offenes Kodieren: *Beim offenen Kodieren werden die Daten durch die Beschreibung von Konzepten in den Daten aufgeschlüsselt, die ein signifikantes Vorkommnis oder ein Ereignis über ein Phänomen darstellen. Es konnten insgesamt 137 Kodes durch diesen Schritt erfasst werden, wie beispielsweise der Kode „Robustheit der Produkte“ der zu der Textstelle *„Unsere PCs brauchen bestimmte Verschraubungen, damit der Schüler nicht sofort alles kaputtmachen kann“ (I4)* zugeordnet wurde.

*Axiales Kodieren:* Beim axialen Kodieren werden die vorliegenden Kodes und dazugehörigen Textstellen auf Zusammenhänge überprüft. Dabei werden Ursachen, Konsequenzen und Interaktionen zwischen den Kodes in Betracht gezogen und zusätzlich kodiert. Durch diesen Schritt konnten acht Ursachen und zehn Konsequenzen kodiert und Kode-Hierarchien gebildet werden. Nach den iterativen Vergleichen der Daten und dem Zusammenführen der axialen Zusammenhänge entwickelten sich 13 Kriterien, die einen maßgeblichen Einfluss auf die Entscheidung haben.

*Selektives Kodieren:* Um über eine bloße Beschreibung der Kriterien hinaus auf eine abstraktere Ebene der Konzeptualisierung zu gelangen (Urquhart et al. 2010) wurde selektives Kodieren angewendet, um die Kriterien zu gruppieren und hierarchische Klassifikationen zu erstellen (Corbin und Strauss 2015). Insbesondere reflektierten wir die Kriterien, indem wir auf den theoretischen Grundlagen des Technology-Organization-Environment-Frameworks (Tornatzky und Fleischer 1990) aufbauten, das Aspekte des Kontexts einer Organisation beschreibt, welche die Adoption von Innovationen beeinflussen. Die drei Kontexte sind (1) technologischer Kontext (technology): umfasst alle technologischen Aspekte, die für die Organisation relevant sind (z. B. technologische Komplexität); (2) organisatorischer Kontext (organization): Eigenschaften und Ressourcen einer Organisation, die einen Einfluss auf die Innovationsadoption haben (z. B. Unterstützung durch das Top-Management); (3) umweltbezogener Kontext (environment): Kriterien wie das regulatorische Umfeld, die Struktur der Branche und das Vorhandensein von Technologieorganisationen. Durch die Anwendung selektiver Kodierung haben wir die identifizierten Kriterien diesen drei Dimensionen zugeordnet, was die Differenzierung der Kriterien unterstützt. Zum Beispiel wurde das Kriterium „Hohe Anschaffungs- und Betriebskosten“ der Dimension „Technologie“ zugeordnet. Tab. 2 stellt die Ergebnisse der Kodierung dar.

**Tab. 2:** Einflusskriterien bei der Technologieauswahl von Schulen

ID	Einflusskriterium	Beschreibung
*Technologische Kriterien*		
K01	Hohe Anschaffungs- und Betriebskosten	Kosten für die Anschaffung und langfristigem Betrieb neuer Technik trotz Förderung
K02	Erschwerte Erfüllung von schulspezifischen Leistungsmerkmalen	Technische Eigenschaften der Produkte, die im Schulumfeld benötigt werden
*Organisationsinterne Kriterien*		
K03	Herausfordernde Kompatibilität mit dem individuellen Schulbedarf	Besondere Bedarfe für unterschiedliche Schulformen und den dortigen Begebenheiten müssen beachtet werden
K04	Unsicherheiten bei der Vereinbarkeit der Technologie mit der individuellen Vision der Lehre	Verständnis der Rolle der Digitalisierung in der Lehre und den zugehörigen Technologien fehlt
K05	Mangelnde Kommunikation	Möglichkeiten zur institutionsübergreifenden Vernetzung werden wenig genutzt
K06	Eingeschränkte IT-Kompetenz der Entscheidungsbeauftragten und Lehrkräfte	Kompetenzen im Umgang mit digitaler Ausstattung sind bisher kaum benötigt worden und rückständig
K07	Fehlender Druck zu Fortbildungen	Qualifizierungsmaßnahmen zu neuen Geräten und digitalen Lehrmöglichkeiten werden nur langsam angenommen
K08	Hoher Personalaufwand	Personalnot beim Schulträger durch Einsparungen
K09	Begrenzte Kompatibilität der Schulgebäude	Zustand der Schulgebäude und Stand der Technik ist nicht überall digitalisierungsfähig
*Umweltbezogene Kriterien*		
K10	Problematische Zentralisierung von Herstellenden	Keine vollständige Marktbetrachtung, sondern Fokus auf bekannte Herstellende
K11	Verteilte und undurchsichtige Zuständigkeiten	Verteilung der Entscheidungskompetenz auf viele Ebenen
K12	Unsicherheiten durch herausfordernden Datenschutz	Mangelndes Verständnis der DSGVO, deren Einhaltung komplex wirkt
K13	Bürokratische Hürden	Staatliche Rahmenbedingungen des Prozesses stellen Herausforderungen dar

## Einflusskriterien des Technologieauswahlprozesses im DigitalPakt

### Technologische Kriterien

#### Hohe Anschaffungs- und Betriebskosten der Technologien

Trotz der initialen Förderung durch das BMBF schreckt der hohe investive Eigenanteil an den Technologiekosten einige Schulträger ab. Die ersten Kommunen haben sogar bereits angekündigt, die Fördergelder überhaupt nicht in Anspruch nehmen zu wollen, da sie *„[…] die Kosten, die danach auf [sie] zukommen scheuen“ (I1)*. Die Interviewpartner:innen schätzen den Nachholbedarf in der schulischen Infrastruktur als zu groß ein, sodass dieser zu teuer und mit weiteren, nur schwer abschätzbaren Folgekosten verbunden ist. Im Rahmen des Prozesses sagt ein Schulträger aus, dass er *„die Gelder erst dann beantragen kann, wenn [er] eine Planung und auch ein Vergabeverfahren gemacht habe“ (I1).* Die Förderung des DigitalPakt ist auf fünf Jahre begrenzt, was im Anschluss daran passiert ist jedoch ungewiss. Wenn ein Schulträger jetzt seine Schulen modernisiert, erhöht er damit auch seine laufenden Kosten durch höheren Verwaltungsaufwand und Geräte, die ersetzt werden müssen. Das hat zur Folge, dass der für die Ausstattung zuständige Schulträger nur dann guten Gewissens eine Förderung beantragen kann, wenn er selbst finanziell gut aufgestellt ist. Auch eine oft notwendige Beratung zur Unterstützung der Technologieauswahl kostet Geld und Zahlungsinstrumente wie Leasing oder Leih-Geräte sind nur in wenigen Bundesländern förderfähig, was die Kluft zwischen den wohlhabenderen und den ärmeren Kommunen weiterwachsen lässt.

#### Erschwerte Erfüllung von schulspezifischen Leistungsmerkmalen

Durch die individuellen Eigenschaften von Schulen werden auch besondere Anforderungen an die Technologien gestellt. In den Segmenten Netzwerke und Hardware sind laut den Befragten vor allem die Faktoren Leistung, Management, Sicherheit, Zuverlässigkeit und Robustheit ausschlaggebend. Die Grundlage für eine Nutzung neuer Technik ist eine leistungsstarke Netzwerkinfrastruktur, die zum Anforderungsprofil von Schulen passt. Dazu gehören primär Router, Access Points, Switches und Firewalls, bei denen es diverse Merkmale zu beachten gilt. So sind beispielsweise Schulen eine komplexe Umgebung mit hoher Nutzungsdichte, weshalb moderne WLAN-Standards und hohe Übertragungsraten nötig sind. Das Management der Geräte sollte zudem zentral über ein Webinterface und einen Managementserver durchgeführt werden, um den Zeitaufwand so gering wie möglich zu halten. Insbesondere weil die IT-Administration in vielen Schulen weiterhin von Lehrkräften durchgeführt wird. Eine weitere geforderte Eigenschaft ist beispielsweise die Möglichkeit mehrere Funknetze auszustrahlen – Multi-SSID. Damit können sich unterschiedliche Nutzergruppen wie Lehrkräfte, Schüler:innen, Verwaltung und Besuchende in das jeweils für sie konfigurierte Netz einloggen.

### Organisationsinterne Kriterien

#### Herausfordernde Kompatibilität mit dem individuellen Schulbedarf

Gemäß des DigitalPakts soll sich die technische Ausstattung der Schule an den zu vermittelnden Inhalten orientieren, weshalb die Erstellung eines Medienkonzeptes für den Förderantrag verpflichtend ist, in dem die pädagogisch-didaktische Nutzung der Geräte erklärt wird. In Deutschland gibt es eine Vielzahl von Schultypen und Schulsystemen und dementsprechend auch unterschiedliche Unterrichtsinhalte. Dazu kommt der sehr individuelle Stand der Technik, der sich stark von Schule zu Schule unterscheidet und ebenfalls einen Einfluss auf die beantragten Technologien hat. So achten Schuladministrator:innen beispielsweise auf gewisse Sicherheitsanforderungen bei der Nutzung, wie Jugendschutzfilter oder eine sichere Netzwerktrennung. Es werden auch flexible Zugänge benötigt, die auf die Dauer einer Unterrichtsstunde begrenzt sind, beispielsweise sollte das Aufheben des Jugendschutzfilters für Schulstunden zur Sexualkunde oder dem dritten Reich unkompliziert und schnell möglich sein. Technologien sollen demnach speziell für Schulen geeignet sein, um nicht nur zu funktionieren, sondern auch praktisch effizient und angepasst an den jeweiligen Unterricht eingesetzt werden zu können.

#### Unsicherheiten bei der Vereinbarkeit der Technologie mit der individuellen Vision der Lehre

Die Vision der Lehre beschreibt die kreative Vorstellung, wie Unterricht zeitgemäß gestaltet werden soll, dazu zählt insbesondere in welcher Form unterrichtet wird, digital, analog oder hybrid, aber auch welche und wie Technologien für eine effektivere Vermittlung von Wissen und demzufolge eine verbesserte Form der Lehre eingesetzt werden können. Die eingesetzten Technologien hängen also stark davon ab, wie die Lehre gestaltet werden soll und welche Vision Schule und Schulträger davon haben. Der erste Schritt einer Schulberatung ist deshalb eine Art Bestandsaufnahme des Lehrerkollegiums und die Feststellung, *„welche pädagogischen Bedarfe“ (I5)* die Lehrkräfte verspüren und ob in ihnen bereits eine Idee und Motivation für zeitgemäßen Unterricht verwurzelt ist. Idealerweise setzt die Vision der Lehre an den Fähigkeiten der Schüler:innen und Lehrkräften an und alle Beteiligten entwickeln sich und ihre Kompetenz im Umgang mit den aktuellen Technologien weiter, sowohl digital als auch analog. So werden in einer modernen Welt moderne Kompetenzen benötigt, welche in der Lehre vermittelt werden sollen, wobei es den befragten Schulträgern nicht darauf ankommt, ob dies mit digitalen oder analogen Medien geschieht. Bei Verantwortlichen in Kommunen, die bei der Ausstattung schon weit fortgeschritten sind, spiegelt sich das auch in ihrer Vorstellung der Lehre wider: *„Meine Vision ist, dass jeder Schüler, der die Schule verlässt, die Kompetenzen für eine digitale Arbeitswelt in einer digitalen Gesellschaft hat. und da ist es völlig [egal], ob der das auf einem Tablet, auf der grünen Tafel oder mit der Schiefer- oder Tontafel macht, aber er braucht diese Kompetenzen für die digitale Welt. Und die digitale Welt ist nicht nur digital, die ist genauso auch analog und hybrid. Aber das ist meine Vision – ein selbstbestimmtes Leben in einer Gesellschaft und Arbeitswelt der digitalen Welt“ (I2).* Um zukunftsorientierte Visionen der Lehre umsetzen zu können, werden geeignete Technologien zur Anwendung aber auch Erprobung gebraucht. Dabei wurde von mehreren Interviewpartnern:innen jedoch angemerkt: *„Ist es nicht absurd, dass die Schulen ein Medienbildungskonzept erstellen müssen, um WLAN zu bekommen? WLAN ist genauso wichtig wie ein Waschbecken in jedem Klassenraum“ (I3)*. Für viele Befragte ist es daher noch unklar, wie sie ihre Visionen der Lehre mit Hilfe des DigitalPakts und verfügbaren Technologien tatsächlich umsetzen können.

#### Mangelnde Kommunikation

Kommunikation ist definiert durch den Austausch und die Vernetzung zwischen Schule, Schulträger, Schulberatung und Land. Bei einem Kommunikationsprozess, der auf so vielen Ebenen abläuft, ist es zwingend notwendig, dass alle Beteiligten das gleiche Ziel verfolgen und gemeinsam an einer Lösung arbeiten. *„Es geht hier um Vernetzung, es geht darum, dass die Schulen teilweise gar nicht wissen, wer ihr Schulträger ist und dass die Schulträger gar keine Ahnung haben, was in den Schulen passiert“ (I3).* Diese Vernetzung soll nun innerhalb kurzer Zeit stattfinden, gestaltet sich aber vielerorts schwierig. *„Dieser Ansatz ist ja, du nimmst alle Schulen, Schulträger, Stiftungen, Medienberatung, was auch immer, alle Stakeholder an einen Tisch und jetzt zeigt sich gerade, die saßen noch nie an einem Tisch“ (I3).* Eine Möglichkeit, die Kommunikation zu verbessern, stellt eine begleitende Beratung dar, die beispielsweise von Landesmedienzentren, unabhängigen Schulberatungen oder Bildungsnetzwerken durchgeführt werden kann. Dies resultiert jedoch in zusätzlichen Kosten.

#### Eingeschränkte IT-Kompetenz der Entscheidungsbeauftragten und Lehrkräfte

Neben dem Nachholbedarf der Infrastrukturen existiert dieser auch an mehreren Stellen bei den Kompetenzen. Die Kommunen werden laut eigener Aussage mit der Lösungsfindung* „alleine gelassen“*. Das Problem dabei beschreibt eine Kommune wie folgt: *„Wenn ich als Stadtverwaltung gestern die Schubkarren für den Friedhofsgärtner gekauft hab und morgen soll ich ein IT-System für 2000 Schüler kaufen, dann habe ich die Kompetenz einfach nicht“ (I2). *Diese Wahrnehmung ist allerdings nicht negativ zu verstehen, denn bisher waren diese Aufgaben schlicht nicht im Anforderungsprofil verankert.

Ein weiterer Aspekt ist die Kompetenz der Lehrkräfte, die mit den Geräten umgehen müssen. Die Bandbreite ist enorm und schwankt von Lehrkräften, die ihre eigenen Lösungen programmieren, bis hin zu solchen, die das Tablet als *„Lineal-Ersatz“ (I1)* verwenden. Es muss nicht jede Lehrkraft eigene Anwendungen programmieren, aber die Kompetenzen, die in der digitalen Welt nötig sind, sollten verstanden und die Technik der Institution sicher beherrscht werden.

#### Fehlender Druck zu Fortbildungen

Die Welt wird zunehmend digitaler und befindet sich permanent im Wandel. Die Lehre allerdings hat sich innerhalb der letzten Jahrzehnte im Vergleich zur freien Wirtschaft nur wenig verändert. Es fehlt an der Stelle der Druck, sich weiterzubilden, der in Wirtschaftsunternehmen vorhanden ist, erläutert ein Netzwerkadministrator einer Gesamtschule, der selbst auch unterrichtet. In der Wirtschaft muss man mit Gehaltseinbußen und der Gefahr des Jobverlusts rechnen, wenn man die angebotenen Fortbildungsveranstaltungen nicht besucht. Das Angebot für Lehrkräfte ist vorhanden, aber wird nicht vollständig ausgeschöpft, da Zeit, Personal und finanzielle Mittel fehlen.

#### Hoher Personalaufwand

Schulträger sind der Dreh- und Angelpunkt im DigitalPakt. Über sie laufen der Beantragungsprozess und in der Regel danach auch Wartung und Support der Geräte. Neben den finanziellen Sorgen sind Personalprobleme ein großer Faktor im Alltag eines Schulträgers: *„über die letzten dreißig Jahre [wurde] in allen Kommunen Personal reduziert, drastisch schlank gespart“ (I1).* Durch den DigitalPakt lässt sich dieses Problem nicht lösen, da keine Gelder für Personal, Service, Wartung und Support abgerufen werden können. Der Schulträger darf nur in Infrastruktur und Geräte investieren, aber wenn kein tragfähiger Rahmen um diese Geräte herum existiert, sinkt deren Effektivität drastisch bzw. ist ein sinnvoller Einsatz eingeschränkt.

#### Begrenzte Kompatibilität der Schulgebäude

Wenn von Modernisierung gesprochen wird, geht damit auch die Frage nach der baulichen Beschaffenheit einher: *„Da ist ein riesen Nachholbedarf. Wir haben ja vor drei Jahren noch diskutiert, ob überhaupt Digitalisierung im Klassenzimmer notwendig ist“ (I3).* Das gilt sowohl für die Stromversorgung der Gebäude als auch für Glasfasernetze oder der generelle Anschluss ans Internet, die überhaupt erst die Voraussetzung darstellen, damit eine Schule WLAN und Endgeräte beantragen kann. Solange die baulichen Beschaffenheiten nicht kompatibel sind, kann auch keine effiziente Modernisierung stattfinden.

### Umweltbezogene Kriterien

#### Problematische Zentralisierung von Herstellenden

Die Ausschreibungen der Schulträger basieren auf einer Einschätzung über die Angebote der Herstellenden. Dabei wird sich hauptsächlich auf große Herstellende fokussiert, welche die gestellten Anforderungen zum besten Preis erfüllen. „*Der, der die letzten Jahre die beste Arbeit geleistet hat auf dem Markt, der ist jetzt der große Gewinner“ (I3) *erklärt eine Schulberatung. Allerdings wird dieser Weg auch hinterfragt und der Blick auf den deutschen Mittelstand gewandt, der dadurch zurückbleibt, denn der wichtigste Aspekt ist, dass das Gerät in der Lehre funktioniert, nicht der Name des Herstellenden.

#### Verteilte und undurchsichtige Zuständigkeiten

Mit diesem Kriterium werden verschiedene Zuständigkeiten und Befähigungen innerhalb des Prozesses betrachtet. Wie bereits erwähnt, müssen bei dem Entscheidungsprozess mehrere Akteure kooperieren, die unterschiedliche Aufgabenbereiche bearbeiten. Dabei stellen sich mehrere Herausforderungen. Die Schulleitung hat selbst kein Geld, ist auch kein:e Eigentümer:in der Schule, muss aber die Ausstattung mit dem Medienkonzept planen und verantwortet die rechtskonforme Umsetzung. Der Schulträger hat, als Eigentümer:in des Gebäudes, die Verantwortung für dessen Ausstattung und die Verwaltung der IT, hat aber in der Regel wenig pädagogischen Hintergrund, um die Bedarfe der Schulen zu verstehen. Das Land, in Form der Kultusministerien, ist zuständig für die strategische Ausrichtung der Lehre, beschäftigt die Lehrkräfte, sagt den Schulträgern aber nicht, welche Ausstattung für diese Lehre nötig ist. Ein komplexes Konstrukt, bei dem viele Interessen kollidieren und personelle wie finanzielle Kapazitäten ausgereizt werden.

#### Unsicherheit durch herausfordernden Datenschutz

Datenschutz und Jugendschutz spielen im geschützten Raum Schule eine große Rolle, insbesondere, wenn neue Technologien Einzug in die Klassenzimmer erhalten sollen. Trotzdem herrscht bezüglich dieses Themas große Unsicherheit. Prinzipiell gibt der DigitalPakt vor, dass förderfähige Geräte DSGVO-konform sein müssen, allerdings gibt es bei der Einhaltung der Verordnung zwei große Probleme. Zum einen gibt es ein Verständnisproblem über die Anforderungen, die eingehalten werden müssen, da die DSGVO gerade für Laien sehr komplex wirkt. Zum anderen herrscht eine unklare Lage um Einzelfälle und es mangelt an klaren Entscheidungsrichtlinien in der Bewertung einzelner Produkte und ihrer Variationen. Durch die Unwirksamkeit des Datenschutzabkommens Privacy Shield darf für eine datenschutzkonforme Lösung keine Verarbeitung personenbezogener Daten über amerikanische Server stattfinden, da das dortige Schutzniveau nicht ausreichend ist. Allerdings sind, zusätzlich verstärkt durch die Corona-Krise, viele installierte Lösungen von den amerikanischen Hyperscalern und Platzhirschen der Branche. *„Die Verunsicherung ist immens, wirklich immens“ (I5)* beschreibt es ein Berater.

#### Bürokratische Hürden

Das Kriterium der Bürokratie beschreibt die Art und Weise, wie Prozessschritte mit Beachtung der staatlichen Vorgaben durchgeführt werden und wirkt sich eher auf die Dauer des Prozesses aus als auf die Entscheidung, welches konkrete Produkt beschafft werden soll. Laut einer Schuladministration beeinflusst es außerdem die Darstellung des zu untersuchenden Problems: *„Die bürokratischen Hürden sind dermaßen hoch, dass wir deswegen noch kein Geld abgerufen haben. Also zu sagen, dass es zögerlich abgerufen wird ist falsch, die Leute schaffen es nicht, das Geld zu holen“ (I4).*

Das bedeutet, selbst wenn der Wille und ein Plan vorhanden sind, gibt es enorme Hürden im Prozess der Beantragung, der den Abfluss der Fördergelder hemmt. Eine Schulberatung stimmt dieser Darstellung zu: *„Dann haben wir zehn Durchschläge, fünf Leute müssen ihr OK dazu geben. Das ist natürlich auch so ein Punkt, den man leider nicht beachtet, der das aber häufig hinauszögert. […] deutsche Bürokratie“ (I5).*

Ein weiterer Aspekt ist die Struktur der Kommunen mit ihren unterschiedlichen Ämtern. Laut Schulträger hat in diesem Umfeld *„jeder […] so seinen eigenen Kopf und jeder hat so seine eigenen Nöte“ (I1).* Das kann beispielsweise dazu führen, dass das Schulverwaltungsamt erst einen Antrag beim Bauamt stellen muss, um ein Kabel verlegen zu lassen. Wenn dort aber die Kapazitäten überlastet sind, dauert das unter Umständen lange.

## Fazit

Das Jahr 2020 hat den Bildungsbereich auf den Kopf gestellt. Eine viel diskutierte Pandemie zwingt auch Schulen bis heute zur Schließung und stellt den vielerorts rückschrittlichen Zustand der Bildungseinrichtungen in den Fokus der Gesellschaft. Eine der wichtigsten Ressourcen Deutschlands, die Bildung, muss sich an die sich weiterentwickelnde Welt anpassen und die damit einhergehenden Chancen nutzen. Der Weg dahin ist allerdings lang und beschwerlich – zu fest ist das traditionelle System in der Breite der Branche verankert, als dass eine Umstellung auf zeitgemäße Lehre in kurzer Zeit vollzogen werden könne. Die Einführung des DigitalPakt Schule setzt daran an, allen Schulen eine Netzwerkinfrastruktur bereitzustellen und sie mit pädagogisch begründeten Geräten auszustatten, sofern sie dafür ein Konzept entwickelt und der verantwortliche Schulträger die Mittel beim Land beantragt. Diese flächendeckende Modernisierung trifft auf einen enorm hohen Nachholbedarf an infrastruktureller Ausstattung und eröffnet zu viele Baustellen auf einmal, um im Rahmen des deutschen Bildungsföderalismus und mit den bürokratischen Hürden schnell zu Ergebnissen zu kommen. Kombiniert mit der ganzheitlichen Veränderung des Bildungssektors gerät dessen Digitalisierung ins Stocken. Unsere explorative Forschung zeigt auf, dass Themen wie Fortbildungen, nachhaltige Wartung und Support, Prozessberatung und Personal ebenso bei Digitalisierung berücksichtigt werden müssen wie WLAN und andere Technologien. Es ist also zwingend eine sozio-technische Perspektive erforderlich, die Mensch und Technik und deren Zusammenspiel betrachtet. Unsere Interviewpartner:innen äußern den Bedarf außerdem an ein Umdenken, hin zu mehr Vernetzung zwischen den Stakeholdern, neuen Ideen für den Unterricht mit digitalen Möglichkeiten und einer nachhaltigen Fokussierung, damit gute Bildung nicht mehr vom Wohlstand der verantwortlichen Kommune abhängt.

Die staatliche Förderung durch den DigitalPakt zeigt, dass zwar deutlich ist, dass in Deutschland bezüglich moderner Bildungseinrichtungen Nachholbedarf besteht, die Umsetzung allerdings wirkt für unsere Interviewpartner:innen überwiegend ineffektiv und die Förderung nicht ganzheitlich genug. Eine Schulberatung sagt: *„Also du musst auch das Raumkonzept der Schule überdenken. Dieser Veränderungsprozess betrifft alles, Kommunikation, Abläufe, Organisation, Verständnis seiner eigenen Rolle“ (I3). *Förderung darf also nicht nur den Kauf von Hardware betreffen, sondern muss die Ganzheitlichkeit des schulischen Umfelds unterstützen.

Keine Forschungsmethode ist perfekt (Myers 2009), daher ist auch die hier durchgeführte explorative Studie mit Limitationen zu betrachten. Diese Arbeit fokussiert sich darauf, den Prozess der Technologieauswahl und dessen Einflüsse im Rahmen des DigitalPakts transparenter darzustellen, da die Besonderheiten des öffentlichen Bereichs bisher wenig erforscht sind. Damit werden jedoch keine konkreten Handlungsempfehlungen für die Zukunft abgeleitet, sondern nur Ideen und Vorschläge auf Basis der Daten der durchgeführten Interviews, der ausgewählten Interviewpartner:innen, und des Wissens der Autoren vorgebracht.

Mit dieser Studie schaffen wir erste Einblicke in die problematischen und verstrickten Ursachen für die (bisher) zögerlichen Abrufe der Fördermittel des DigitalPakts. Insgesamt konnten wir 13 Kriterien identifizieren, welche den Technologieauswahlprozess beeinflussen. Dabei sind nicht nur technologische (bspw. Kosten), sondern auch organisationsinterne (bspw. fehlendes Knowhow) und umweltbezogene Kriterien relevant (bspw. verteilte und undurchsichtige Verantwortlichkeiten). Basierend auf unserer Studie sollte zukünftige Forschung nicht nur Handlungsempfehlungen für alle relevanten Akteure ableiten, um den Technologieauswahlprozess zu unterstützen. Vielmehr sollten konkrete Mittel zur Lösung der bestehenden Probleme identifiziert und umgesetzt werden. So können bspw. neue Datenschutz-Zertifizierungen (gemäß Art. 42 DSGVO) für Technologien entwickelt werden, um Transparenz über und Vertrauen in Angebote am Markt zu schaffen und den Auswahlprozess zu vereinfachen (Maier et al. 2019).
